# Stem growth characteristics of high yielding *Miscanthus* correlate with yield, development and intraspecific competition within plots

**DOI:** 10.1111/gcbb.12610

**Published:** 2019-03-18

**Authors:** Paul R. H. Robson, Iain S. Donnison, John C. Clifton‐Brown

**Affiliations:** ^1^ Institute of Biological, Environmental and Rural Sciences (IBERS) Aberystwyth University Gogerddan, Aberystwyth Wales United Kingdom

**Keywords:** bioenergy, energy crops, growth curves, *Miscanthus*, modeling, yield

## Abstract

High yielding perennial grasses are utilized as biomass for the bioeconomy and to displace fossil fuels. Many such grasses, including *Miscanthus*, are largely undomesticated. The main *Miscanthus* crop is a naturally occurring hydrid *M. × giganteus* (*Mxg*). All above ground biomass from *Miscanthus* is harvested. Stem traits correlate strongly with yield and therefore understanding the seasonal progression of stem growth should identify routes for improved yield. If such studies utilized high yielding commercial genotypes growing in plots the conclusions are likely to be more commercially relevant. Stem elongation was measured from five high yielding genotypes, 10 plants per plot from 20 plots in a replicated field trial over 4 years. Richards growth function produced an accurate fit to stem elongation. Differentials, double differentials and integrals of the parameterized function produced six growth characteristics, describing growth rate, timing and duration of the logarithmic growth phase and area under the growth curve. Maximum growth rate was correlated with yield and compensatory interactions were identified, for example plants with higher maximal growth rates had shorter durations of logarithmic growth. Plant position within plots of lower yielding genotypes did not affect growth characteristics but had a significant effect on late season growth characteristics in higher yielding genotypes. Two high yielding genotypes were compared over 3 years and growth parameterized using four different factors. The inverse correlation between maximum growth rate and duration of logarithmic growth was consistent across years and factors in both genotypes except when parameterized using temperature and only in *Mxg*. This suggested that different limitations to growth were exerted on the two genotypes which may help explain the exceptional performance of the *Mxg* genotype. We discuss the implications of the identified complex interactions in growth characteristics for approaches to maximize seasonal yield in perennial biomass crops.

**Abbreviations**
AUC: area under the curve (from integral)
Cd: degree days
DayMaxGR: day of maximum growth rate (from first differential)
Duration: duration of logarithmic growth phase
EndLogG: end of logarithmic growth phase (from second differential)
GI: global irradiation
Jd: Julian day
MaxGR: Maximum growth rate (from first differential)
*Mxg*: *Miscanthus* *×* *giganteus*
PTQ: photothermal quotient
StartLogG: start of logarithmic growth phase (from second differential)

## 1 INTRODUCTION

Dedicated biomass crops provide a suitable alternative to fossil fuels and waste biomass products, the latter being rarely available at sufficient scale. Dedicated perennial biomass crops such as *Miscanthus* have a high energy ratio and thus high potential greenhouse gas savings when compared to alternatives such as gas and coal (reviewed in McCalmont et al., 2017). When crops are grown for other purposes such as food it is more acceptable to utilize high energy in production but this is counterproductive in biomass crops. Other considerations in growing biomass crops include competition for land and utilizing marginal land that is not economic for food production (Valentine et al., 2012). Improvements in intrinsic seasonal biomass accumulation will improve energy ratios, land use efficiency and the economics of biomass crops. However the traits that have been used to domesticate conventional food crops are likely to be different to the most important traits for domesticating biomass crops (Sang, 2011).

All above ground biomass of the *Miscanthus* plant is harvested and yield is the integral of many traits acting across a long growth season. The many traits that contribute to yield and yield quality in *Miscanthus* include morphological traits such as canopy height (Jeżowski, 2008; Jones, Finnan, & Hodkinson, 2015), phenological traits such as early season emergence, developmental traits such as flowering and senescence (Fonteyne et al., 2018; Jensen et al., 2011; Robson, et al., 2011), and compositional traits (Da Costa et al., 2014; Jorgensen, 1997). In a study of several aboveground traits canopy height was highly correlated with yield although there may be other confounding interactions for example with stem number (Robson et al., 2013b).

Stem length is a simple measurement requiring no specialized equipment. When measured over time the progression of stem length can be modeled by parameterized functions (Richards, 1959). Standard derivatives of growth functions may be used to produce growth characteristics that describe both the speed of growth and the timing and duration of growth (Shi et al., 2016). Such functional approaches have been used in QTL analysis which demonstrated that the use of functional data improved the power to detect QTL (Wu & Lin, 2006).

We were interested to discover if functional data could be used to identify the growth characteristics of higher yielding *Miscanthus* crops. We further wanted to test if these characteristics were consistent across years and to what extent they were impacted by intraspecific competition and meteorological parameters. Several studies have reported genotypic variation across a range of traits and highly diverse germplasm (Clark et al., 2016; Zub & Brancourt‐Hulmel, 2010). While such studies are highly informative of the biology, due to the inclusion of low yielding genotypes, they may over emphasize the potential usefulness of the information in achieving high yield. We have focused on high yielding *Miscanthus* from a commercial breeding programme and included the current commercial standard *M. × giganteus *(*Mxg*). We proposed that growth curves would be highly informative for yield in this crop. Because the entire above ground biomass is harvested the stem growth curve reflects the accumulation of harvested product. *Miscanthus* represents an excellent system to study growth curves because it is a large plant that grows over a long season and therefore errors associated with small measurements and short growth periods are less significant. *Miscanthus* is perennial and therefore allows investigation of how growth dynamics are linked across years.

Here we used high yielding *Miscanthus* genotypes growing at commercial planting densities in a replicated field trial and measured stem length across multiple years. The data described here represent mature plants during the third, fourth and fifth years of growth, as years one and two were considered to be of limited predictive value for long‐term yield projections (Lewandowski, Clifton‐Brown, Scurlock, & Huisman, 2000). Five genotypes were assessed including *Mxg*, in one year to determine the associations between growth characteristics and yield. Two of the highest yielding genotypes were measured over a further three years to test how consistent were the growth characteristics when expressed as a function of different meteorological factors.

## 2 MATERIALS AND METHODS

The trial site was established on a sloping field (52°26′N, 04°01′W) near Aberystwyth on the west coast of Wales. The soil is classified as a dystric cambisol and a dystric gleysol depending on spatial variation in drainage (FAO, 1988) with a stone fraction (particles >2 mm) of approximately 15% (0–30 cm soil layer). Soil texture was 18% clay, 24% silt and 58% sand. Four randomized blocks of five 25 m^2^ (6.67 m × 3.75 m) plots were separated by an equivalent of one planting row, and blocks by 3 m paths. The 20 plots in the trial were oriented across a gentle slope which declined by 3 m from upper edge of the first block to the lower edge of the fourth block. Fifty plants per plot were planted resulting in a planting density of two plants m^−2^ which has been used as a standard for evaluating *Miscanthus* over the past 20 years (Atkinson, 2008). The four novel *Miscanthus* genotypes, selected from interspecific *M. sacchariflorus* *×* *M. sinensis* crosses (Hy1‐4) made by Martin Deuter, were cloned using *in vitro* tillering and planted as bare rooted plug plants (plug compost was washed out). The control genotype *M. × giganteus* was planted at two rhizomes m^−2^ in the same week.

The planted area was sprayed with Atrazine (3 L ha^−1^) on 5 April 2005 to kill a ryegrass cover crop. Plants were planted by hand into weed free soil between 20 and 28 May 2005. Plants were watered at planting to ensure good soil to plant hydraulic contact to minimize transplanting losses, no further weeding and no fertiliser treatments were applied. Where plants did not survive transplanting, replacements were made in the first 6 weeks. Plots were harvested each spring (between 15 Feb and 15 March depending on local weather conditions) to determine yield and moisture content. The two longest border rows, of 10 plants each, were excluded from the harvest sample area, leaving 15 m^2^ out of the total plot area of 25 m^2^. In common with breeders plots used for cereals, six out of 30 harvested plants were ‘border plants’. The fresh weight of 15 m^2^ per plot was weighed to the nearest 100 g. From this a random subsample of at least 150 g fresh weight (approximately five stems) was weighed fresh and dried at 60°C until constant weight. Moisture content was calculated from the subsample weights, and used to calculate the bulk plot dry matter yield equivalent in oven‐dried tonnes per hectare. The correlations between yield data and averaged growth characteristics calculated from stem elongation data were tested as described below.

### 2.1 Growth measurements

Stem elongation was measured approximately fortnightly throughout the growth season usually from May to October. The middle rows of 10 plants (from the 10 × 5 plots) were measured from each of 40 plots producing 7,020 stem measurements. The plots were oriented approximately east‐to‐west, with Plant 1 being at the approximately east facing and upper edge of the slope and plant 10 at the approximately west facing lower edge of the plot. Stem elongation was measured as the length of stem from the ground along the length of the longest stem to the point on the stem that was subtended by the ligule of the youngest differentiated leaf. Because *Miscanthus* is multi‐stemmed and it was not always obvious which was the longest stem, a sample of the three longest stems was measured and the length of the longest recorded. Complete plots were established in 2005; however, after the crop had matured and the first measurements made in 2008, four plants were missing from the central measurement transects, therefore 196 plants were measured and growth curves parameterized as described below. After parameterization of the 2008 data the growth curves of two plants could not be fully parameterized due to the lack of an upper asymptote and these plants were removed from subsequent analysis. With these exceptions 10 plants per genotype per block were measured and growth characteristics estimated from each plant. Where appropriate growth characteristics were averaged as described below.

### 2.2 Meteorological factors

Daily climate data for the trial site were obtained from the Gogerddan weather station (52°25′N, 04°01′W), 500 m from the trial site. Data were recorded using a datalogger (Type CR10, Campbell, Leicestershire, UK). Heat accumulation, in degree days (°Cd), was calculated on a daily time step above a threshold temperature of 10°C, using equations described by McVicker (1946) from daily maximum and minimum air temperatures. Daily incident global irradiance (GI) values in MJ m^−2^ day^−1 ^were calculated for the site from the mean daily radiation received by a pyranometer (Skye Instruments, Powys, UK) attached to the same meteorological station. Daily global irradiation was accumulated throughout the year except if the mean daily temperature <10°C then that days accumulated GI = 0. Photothermal quotient (PTQ), expressed as MJ m^−2^ day^−1^ °C^−1^, was calculated as the ratio of total solar radiation in MJ m^−2^ day^−1^ to the mean daily temperature minus a base temperature (10°C for *Miscanthus*). PTQ was accumulated throughout the year except if the mean daily temperature <10°C then that days accumulated PTQ = 0.

### 2.3 Data analysis and modeling

All analysis was completed using R (R Core Team, 2015) with some bespoke scripts for fitting equations to data. Functions were fit to individual plant data using the ‘nlsLM’ function from the package ‘minpack.lm’. Two functions were used in custom scripts: a three parameter sigmoid function and a four parameter Richards growth function. The custom scripts were compared with a beta function script available for R (Shi et al., 2016).

Data were analyzed for normality using the Shapiro Wilkes test from the R package ‘nortest’. Correlations used the ‘cor’ library and either the ‘pearson’ method to calculate the parameteric Pearson’s product‐moment coefficient of correlation (*r*) or the ‘spearman’ method to calculate the rho statistic for ranked data. The significant differences between the means of groups were compared by ANOVA. This was performed using the R aov function and the Tukey’s HSD test from the R library ‘Agricolae’ (Mendiburu, 2017).

The three parameter Sigmoid and four parameter Richards growth functions (Richards, 1959) used are shown in Equations 1 and 2 where *x* is the explanatory variable and *a*, *b*, *c* and *d* parameters of fit.

(1) $$\frac{a}{1+\exp^{-b*(x-c)}}$$

(2) $$\frac{a}{1+b*\exp^{({-c*x)}^{\left(\frac{1}{d}\right)}}}$$

The parameters derived from individual plant data and the Richards function were used for further study. Parameterized functions were differentiated to identify the maximum growth rate (MaxGR) and when MaxGR occurred was estimated to the nearest Julian day (DayMaxGR). When other explanatory factors were used to calculate growth parameters the ‘Day’ prefix was kept for consistency, although the factors were not days values were accumulated daily. The second differential was used to estimate the start and end of the logarithmic growth phase (StartLogG) and (EndLogG) respectively and the difference between the two was calculated to be the duration of the logarithmic growth phase (Duration). Duration was further subdivided for analysis across three years by calculating the difference between StartLogG and DayMaxGR and the difference between DayMaxGR and EndLogG. The final growth characteristic calculated was the integral of the curve (AUC).

### 2.4 Inter‐year comparison

For the first year in which measurements were made all five genotypes were measured. Using data from the first measured growth season one high yielding genotype (Hy2) and the commercial type (*Mxg*) were chosen for further study across three subsequent years. To examine the effect of different meteorological explanatory variables, growth curves were fit to data from the two genotypes growing in each block, averaged from 10 plants within the block, and from three consecutive years. First the parameters of the Richards growth equation were calculated using one of four explanatory variables, Julian day (Jd), °Cd, GI and PTQ (as described above) and the parameterized equations were used to calculate the level of explanatory factor at two stem lengths for each plant. The stem lengths chosen were a stem length close to emergence that was within the range of measured stem length data (15 cm), that is, did not require interpolation, and a stem length in the log growth phase (150 cm). The values of the explanatory variables at the two stem lengths were compared across the three years to see how consistent these factors were using repeated measures ANOVA of a linear model with genotypes as a random factor using the ‘nlme’ package in R (R Core Team, 2015). Calculations of F‐statistic and Student’s *t* test were performed using either Excel or the ‘ez’ package in R.

The characteristics of individual plant growth curves from each year were calculated as described above for each of four different explanatory factors. Using the parameters of the growth equation for each year the explained characteristics were calculated and compared between years using the lm function or were compared using correlation as described above. The parameters from the four blocks and three years were tested for normality and approximately two‐thirds were found to violate the assumption of normality at *α* = 0.05 and therefore to perform correlations the data were ranked and correlation compared using Spearman’s rho statistic.

## 3 RESULTS

### 3.1 Curve fitting

Custom scripts using sigmoid and Richards growth functions (Equations (1) and (2)) produced low sum of squares error terms. Visual inspection of fitted curves using the beta function (Shi et al., 2016) showed a mixture of good and poor fit whereas the other two functions were more consistent and approximated the data points accurately. The Sigmoid and Richards functions produced similar accuracies of fit but Richards function allowed nonsymmetrical fit to sigmoid data, therefore, despite requiring one additional parameter, was chosen for further study.

### 3.2 Growth characteristics

The characteristics of seasonal growth, derived from parameterized functions for each plant, all had a significant genotypic effect. There was no significant effect of Block on any of the growth characteristics. Genotypes Hy3 and Hy4 formed a separate group when analyzed post hoc using Tukey’s HSD test, which had significantly later StartLogG, DayMaxGR, and EndLogG (Table 1). Duration was significantly different between Hy3 and Hy4. Duration in Hy4 was short and similar to *Mxg* and Hy1 which had the shortest Duration; Hy3 was longer in Duration and more similar to Hy2. The maximum growth rate (MaxGR) was significantly different between most genotypes; *Mxg* and Hy1 were assigned to the same group *post hoc* and had the highest MaxGR, the other three genotypes were significantly different with MaxGR decreasing in the order of Hy2, Hy4 to the lowest values from Hy3. The log growth phase ended significantly later in Hy3 and Hy4 than in the other three genotypes. Hy3 entered log phase significantly earlier than Hy4 but the maximum growth rate was significantly lower in Hy3 (Table 1).

**Table 1:** Seasonal characteristics of stem elongation from five high yielding *Miscanthus* genotypes grown in field plots from one growth year

Genotype	MaxGR	Day MaxGR	StartLogG	EndLogG	Duration
Hy1	2.8 ± 0.1 (a)	179.4 ± 2.7 (b)	151 ± 2.1 (bc)	207.7 ± 3.7 (c)	56.6 ± 2.4 (c)
Hy2	2.3 ± 0.1 (b)	183.1 ± 2.3 (b)	146 ± 2.2 (c)	220.3 ± 3.7 (b)	74.2 ± 3.9 (ab)
Hy3	1.6 ± 0.1 (d)	216.7 ± 7.0 (a)	160.9 ± 7.5 (b)	237.8 ± 8.9 (a)	76.9 ± 14.1 (a)
Hy4	2.0 ± 0.1 (c)	214.5 ± 6.3 (a)	172.8 ± 4.8 (a)	237.5 ± 8.4 (a)	64.6 ± 11.4 (bc)
*Mxg*	2.8 ± 0.2 (a)	181.4 ± 3.1 (b)	148.5 ± 2.0 (c)	213.8 ± 3.8 (bc)	65.3 ± 3.2 (bc)

The genotypes were four commercial hybrids Hy1 to Hy4 and the widely grown commercial standard *M. × giganteus* (*Mxg*). The characteristics calculated from stem growth curves were: maximum growth rate (cm/day) (MaxGR) and the day on which this was noted (DayMaxGR), the start and end of the rapid growth phase (StartLogG and EndLogG respectively) and the duration of the rapid growth phase (Duration). Tukey’s HSD statistic for significant differences between groups is reported in brackets (block values were averages of 10 stems from four blocks, *n* = 4).

### 3.3 Plant position and growth characteristics

There was no significant effect of plant position on StartLogG within the 10 plant transects across each plot; however, all other growth characteristics were significantly affected, as reported by pairwise ANOVA. There was no significant interaction between plant position and block therefore plants were grouped across blocks and examined as individual genotypes. None of the plant growth characteristics from the lowest yielding genotypes Hy3 and Hy4 were significantly affected by position within the transect. However MaxGR, DayMaxGR, EndLogG and Duration were all significantly affected by plant position in Hy2 and *Mxg* and MaxGR and Duration were significantly affected by plant position in Hy1 (data not shown).

### 3.4 Growth characteristics and yield correlations

There was a significant and highly negative correlation between MaxGR and Duration (*R* = −0.73, *p* = 2.9 × 10^−4^) (Table 2). Duration was significantly and positively correlated with EndLogG (*R* = 0.65, *p* = 0.002) but not significantly correlated with DayMaxGR or StartLogG. DayMaxGR was significantly and similarly correlated with both the start and end of the logarithmic growth phase (*R* = 0.86 and 0.94; *p* = 9.5 × 10^−7^ and 1.2 × 10^−9^ respectively). DayMaxGR was compared to the midpoint between StartLogG and EndLogG. Variances were equal comparing the midpoint and DayMaxGR for each genotype (*F*‐test) and the means were significantly different only for genotypes Hy3 and Hy4 (Student’s *t* test) at an alpha of 0.1 (*p* = 0.051 and 0.091 respectively). DayMaxGR was approximately 16 and 9 days later than the midpoint calculated from the second differentials from Hy3 and Hy4 respectively. The DayMaxGR and midpoint between StartLogG and EndLogG were not significantly different in the other three higher yielding genotypes differing by on average 0.1 to 0.2 days.

**Table 2:** Growth and biomass yield correlations with five high yielding *Miscanthus* growing in field plots

Variables	1	2	3	4	5	6
1. MaxGR	—					
2. DayMaxG	−0.88***	—				
3. StartLogG	−0.57**	0.86***	—			
4. EndLogG	−0.93***	0.94***	0.72***	—		
5. Duration	−0.73***	0.40 ^.^	−0.05	0.65**	—	
6. MC	−0.06	−0.07	−0.39 ^.^	−0.001	0.43***	—
7. Yield	0.82***	−0.92***	−0.77***	−0.84***	−0.36	−0.09

Variables are: maximum growth rate (MaxGR); day at which maximum growth rate was recorded (DayMaxGR), start of logarithmic growth (StartLogG), end of logarithmic growth (EndLogG), duration of logarithmic growth (Duration), moisture content of harvested biomass (MC), weight of harvested biomass in oven dried tonnes ha^−1^ (Yield). Significance level is denoted by symbol: (***, 0.001; **, 0.01; *, 0.05; ****, 0.1).

Harvested yield was higher in crops with a higher MaxGR (*R* = 0.82, *p* = 9.0 × 10^−6^), but if the MaxGR occurred later in the season the yield was lower (DayMaxGR, *R* = −0.92, *p* = 8.9 × 10^−9^). There were similar negative correlations between yield and StartLogG and EndLogG (*R* = −0.77 (*p* = 6.1 × 10^−5^) and −0.84 (*p* = 3.7 × 10^−6^) respectively). Duration was moderately correlated with moisture content at a moderate level of significance (*R* = 0.43, *p* = 0.06), all other correlations with moisture content were low and not significant (Table 2).

### 3.5 Inter‐year comparisons

**Figure 1:**
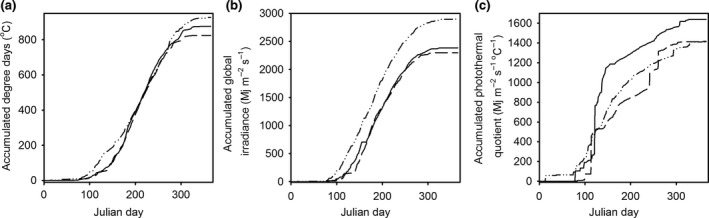
Accumulation of meteorological parameters (a) degree days, (b) global irradiance and (c) photothermal quotient, used to calculate growth characteristics across three study years (2009–2011) at a field site close to Aberystwyth, UK. Solid line (2009); dashed line (2010) and dashed + dotted line (2011)

The parameterized equations from stem measurements of the commercial type *Mxg* and the high yielding genotype Hy2 were used to estimate stem growth characteristics across three different growth years as determined by Julian day (Jd) and three meteorological factors (Figure 1). Early stem height (15 cm) and later stem height (150 cm) showed similar but not identical trends (Table 3). The number of Jd to 15 cm was similar comparing the two genotypes and the repeated measures model showed a significant difference between years. The number of Jd to 150 cm was significantly different and later in Hy2 than *Mxg*. The repeated measures model showed no significant difference between years in the accumulated degree days (°Cd) to 15 cm stem length and this was the only comparison where a non‐significant effect of year was found. The effect of year was significant in comparing the °Cd to 150 cm stem length and was calculated to occur at a lower accumulated degree day in *Mxg* than in Hy2 (Table 3). Differences between genotypes were either not significant or trends were not consistent across the 3 years when either global irradiance or photothermal quotient was included as explanatory variables (Table 3).

**Table 3:** Estimated days and meteorological factors accumulated to early and late stem lengths from two high yielding genotypes of *Miscanthus* growing in three consecutive growth years of a mature plot

Geno‐type	Year	Jd 15 cm	Jd 150 cm	°Cd 15 cm	°Cd 150 cm	PTQ 15 cm	PTQ 150 cm	GI 15 cm	GI 150 cm
Hy2	2009	145 (1.9)	201 (2.0)	88.4 (6.3)	393 (10.7)	1,102 (28.3)	1,292.9 (6.1)	449.8 (36.4)	1,321.7 (32.7)
Hy2	2010	143.3 (1.4)	199.7 (1.9)	79.1 (6.5)	367.6 (11.7)	549 (8.7)	843.2 (4.1)	231.1 (30.4)	1,327.2 (23.6)
Hy2	2011	115.2 (3.7)	191.2 (2.6)	85.1 (13.6)	350.9 (12.9)	404.7 (38.9)	988.9 (11.7)	353.4 (57.9)	1,659.5 (49.3)
*Mxg*	2009	144.5 (2.1)	193.3 (2.8)	87.9 (6.4)	352.1 (15.6)	1,092.9 (30.5)	1,270.6 (8.6)	442.4 (37.9)	1,203.6 (45.7)
*Mxg*	2010	145.3 (1.3)	196.6 (2)	88.6 (4.9)	348.1 (12.3)	561.5 (10.3)	836.6 (4.3)	276.8 (28.6)	1,289 (25)
*Mxg*	2011	119 (4.3)	187.6 (2.0)	99.6 (14.9)	332.8 (10.7)	441.7 (40.7)	971.6 (10.3)	412.6 (64.9)	1,588.2 (43.1)

The genotypes were commercial hybrid Hy2 and the widely grown commercial standard *M. × giganteus* (*Mxg*). Factors are Julian day (Jd), accumulated degree days (°Cd), accumulated photothermal quotient (PTQ) and accumulated global irradiance (GI). Values are averaged from 10 plants per block and calculated means are from four blocks, standard error is shown in brackets (*n* = 4).

The six growth parameters were calculated, as stated above, from the parameterized equations derived using four explanatory variables across three years, two genotypes and four blocks. For all four factors *Mxg* had a higher AUC and MaxGR than Hy2. The day at which MaxGR occurred was either not significantly different (2009) or was later (2010 and 2011) in *Mxg*. Other comparisons between years, parameters and genotypes were more complex involving different seasonal times and therefore levels of factors, so for simplicity the parameters from the growth curves were compared across years and genotypes using correlation coefficients. The aim was (1) to identify if meteorological data produced more consistent trends across years, as was the case for °Cd to 15 cm stem length, which might suggest the nature of the factors that better explained elongation growth and if so (2) to identify if there were genotypic differences in which factors better explained such growth.

Growth characteristics were parameterized from each plant and averages from each Block tested for correlations across multiple years. The parameters changed between growth years and there was a significant effect of genotype on all growth characteristics. To further examine if the growth characteristics changed similarly in different years in the two high yielding genotypes correlation coefficients between different growth characteristics were calculated. A higher coefficient of correlation indicated growth characteristics were affected similarly by variation in the explanatory factors between years.

**Figure 2:**
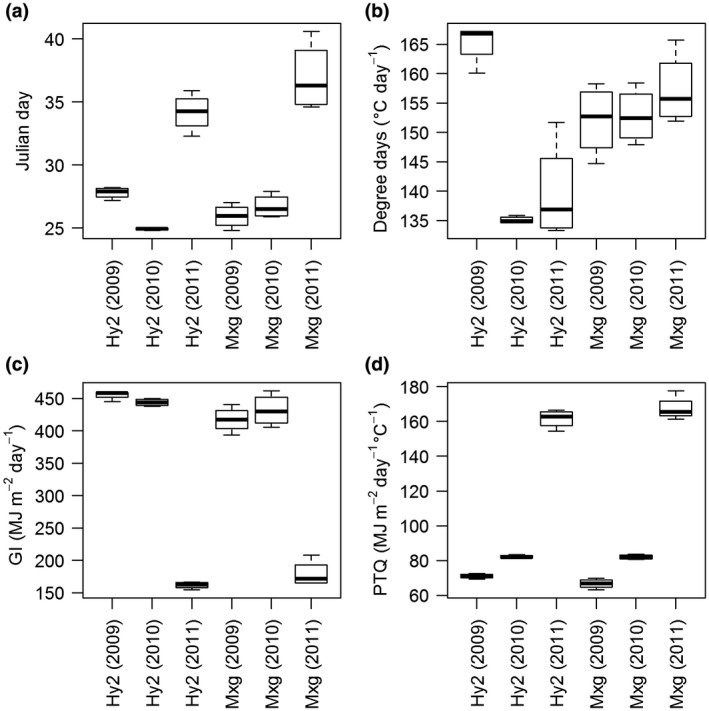
Value ranges for the difference between the factor value at which maximum growth rate was calculated and factor value at the end of the logarithmic growth phase calculated across three growth years, two genotypes Hy2 and *M. × giganteus* (*Mxg*) and four factors Julian day (a), degree days (b), global irradiance (c) and photothermal quotient (d)

MaxGR and Duration in Hy2 were highly and negatively correlated, as was the case in the five genotype studies (Table 2), when derived using all four factors. MaxGR and Duration in *Mxg* were highly negatively correlated when derived using Jd, GI and PTQ but there was no significant correlation when derived using °Cd (Table 4). Duration is calculated from the second differentials defining the start and end of the logarithmic growth phase. The correlations across years between MaxGR and StartLogG were similar in the two genotypes, whereas that between MaxGR and EndLogG differed significantly only in *Mxg* when °Cd was used as the explanatory variable. When °Cd was used as a factor MaxGR was significantly and highly correlated with EndLogG in *Mxg* but this correlation was negative and not significant in Hy2 (Table 4). To investigate this further for each explanatory factor the period of Duration was split in two as the difference between DayMaxGR and either StartLogG or EndLogG. The difference between StartLogG and DayMaxGR varied significantly across years when each genotype was tested with each of the four factors according to a repeated measures ANOVA model. In a similar exercise the difference between DayMaxGR and EndLogG varied significantly across years in both genotypes and all four explanatory factors except when calculated from °Cd (Figure 2). There was no significant difference in the accumulated degree days between DayMaxGR and EndLogG in *Mxg*; whereas in Hy2 this value varied significantly particularly in 2009 (Figure 2b).

**Table 4:** Correlations between selected ranked growth curve characteristics from two high yielding *Miscanthus* genotypes across three growth years

Geno.	Var.	First char.	Second char.	rho	*p*‐value	Var.	rho	*p*‐value
*Mxg*	Jd	MaxGR	Duration	−0.96	9.5 × 10^−7^	GI	−0.92	1.9 × 10^−5^
*Mxg*	Jd	MaxGR	StartLogG	0.76	3.9 × 10^−3^	GI	0.60	3.9 × 10^−2^
*Mxg*	Jd	MaxGR	EndLogG	−0.84	6.4 × 10^−4^	GI	−0.91	4.2 × 10^−5^
*Mxg*	Jd	StartLogG	Duration	−0.68	0.02	GI	−0.70	1.1 × 10^−2^
*Mxg*	Jd	EndLogG	Duration	0.92	2.8 × 10^−5^	GI	0.90	6.0 × 10^−5^
*Mxg*	Jd	StartLogG	EndLogG	−0.49	0.10	GI	−0.49	0.11
Hy2	Jd	MaxGR	Duration	−0.87	2.0 × 10^−4^	GI	−0.53	7.5 × 10^−2^
Hy2	Jd	MaxGR	StartLogG	0.72	8.5 × 10^−3^	GI	0.46	0.13
Hy2	Jd	MaxGR	EndLogG	−0.81	1.6 × 10^−3^	GI	−0.88	1.5 × 10^−4^
Hy2	Jd	StartLogG	Duration	−0.90	6.6 × 10^−5^	GI	−0.85	4.2 × 10^−4^
Hy2	Jd	EndLogG	Duration	0.82	1.2 × 10^−3^	GI	0.61	3.6 × 10^−2^
Hy2	Jd	StartLogG	EndLogG	−0.52	8.5 × 10^−2^	GI	−0.42	0.17
*Mxg*	°Cd	MaxGR	Duration	0.03	0.91	PTQ	−0.98	3.1 × 10^−8^
*Mxg*	°Cd	MaxGR	StartLogG	0.95	2.0 × 10^−6^	PTQ	0.45	0.14
*Mxg*	°Cd	MaxGR	EndLogG	0.74	5.8 × 10^−3^	PTQ	0.36	0.25
*Mxg*	°Cd	StartLogG	Duration	0.15	0.65	PTQ	−0.49	0.12
*Mxg*	°Cd	EndLogG	Duration	0.59	0.04	PTQ	−0.36	0.26
*Mxg*	°Cd	StartLogG	EndLogG	0.85	4.2 × 10^−4^	PTQ	0.90	8.4 × 10^−5^
Hy2	°Cd	MaxGR	Duration	−0.79	2.2 × 10^−3^	PTQ	−0.94	3.9 × 10^−6^
Hy2	°Cd	MaxGR	StartLogG	0.81	1.4 × 10^−3^	PTQ	0.50	0.10
Hy2	°Cd	MaxGR	EndLogG	−0.38	0.23	PTQ	0.45	0.14
Hy2	°Cd	StartLogG	Duration	−0.52	0.08	PTQ	−0.48	0.12
Hy2	°Cd	EndLogG	Duration	0.73	7.4 × 10^−3^	PTQ	−0.41	0.18
Hy2	°Cd	StartLogG	EndLogG	0.05	0.88	PTQ	0.95	2.0 × 10^−6^

Geno. = two high yielding *Miscanthus* genotypes (*M. × giganteus* (*Mxg*) and Hy2). Four explanatory variables (Var.): Julian days (Jd), accumulated degree days (°Cd), accumulated global irradiance (GI) and accumulated photothermal quotient (PTQ). Growth characteristics (char.): maximum growth rate (MaxGR), start of logarithmic growth (StartLogG), end of logarithmic growth (EndLogG), duration of logarithmic growth (Duration).

## 4 DISCUSSION

Yield in *Miscanthus* comprises all above ground biomass and therefore we hypothesized that the modeling of key parameters that describe growth curves would identify how seasonal development interacts with yield. Robson et al., (2013a) showed higher yield was associated with longer canopy durations as measured by the composite duration trait including early season light interception by leaf and senescence. Zub et al., (2012) measured early stem elongation and showed that later growth and faster growth rates were associated with higher yield. We demonstrate that in agreement with Zub et al. (2012) genotypes with high maximum growth rates (MaxGR) tended to have higher yields but there was an inverse correlation between Duration and MaxGR (*R* = −0.55, *p* = 0.01). It has been demonstrated that extending the growth season via delayed flowering results in increased yield (Jensen et al., 2013). Our results demonstrate a more complex interaction between growth rate, duration and yield that may be explained by a seasonal ceiling to yield or net primary productivity that limits yield within a system (Field et al., 2008). The ceiling in growth may not necessarily be a function of the ecosystem but could be due to the attainment of a sink‐source flux that triggers for example the reproductive pathway (Moghaddam & Van den Ende, 2013; Turnbull, 2011) reducing further elongation growth and yield accumulation. Previous experiments (Robson et al., 2013b; Zub et al., 2012) have used highly diverse genotypes including low yielding ones that may emphasize the advantages of longer durations or faster growth rates. In this study utilizing high yielding commercial genotypes that are well adapted to local conditions and grown at commercial planting densities has allowed us to identify more commercially relevant seasonal interactions. The implications of these findings in superior commercial genotypes are that if mechanisms are applied to improve yield, such as more efficient photosynthesis, that do not take account of the ceiling in yield then no net improvement will be achieved, the plants will simply reach maximal yield faster.

The Richards function allowed non‐symmetrical sigmoid fit to stem elongation data. The maximum growth rate in high yielding *Miscanthus* genotypes occurred at the midpoint between the start and end of the logarithmic phase whereas in lower yielding *Miscanthus* this occurred after the midpoint. This result suggests that the logarithmic growth phase was curtailed in the lower yielding genotypes and without a developmental or other curtailment of growth the lower yielding *Miscanthus* would be longer in the logarithmic phase post maximum growth. The comparison of midpoints may give us insight into the extent of local adaptation within genotypes with the higher yielding plots of genotypes including *Mxg* able to complete the logarithmic duration, as indicated by overlapping midpoints, and thus greater maximal net potential productivity. Different genotypes will be more or less well adapted to a particular experimental system. Important factors determining the extent of adaptation may include seasonal variation in meteorological conditions and planting density.

Julian day is aliased to some extent with meteorological data such as temperature and irradiation, but we tested if there was sufficient variation between years to determine if stem elongation modeled with different explanatory meteorological variables could be used to identify which factors best explained elongation growth. We were able to demonstrate a consistent accumulation of thermal time to early stem elongation (15 cm) but not to later (150 cm) and this occurred at a lower accumulated thermal time in *Mxg*. Early season growth is expected to be primarily driven by temperature and not be limited by light because the canopy is not closed. Early growth may utilize stored carbohydrate in rhizome (Beale & Long, 1997) and; therefore, early growth would be less dependent on light. As the canopy closes light and temperature may have a significant and combined effect. In seed and grain crops yield has a moderate correlation with photothermal quotient when applied across the flowering period (Fischer, 1985; Nalley et al., 2009; Poggio et al., 2005). In perennial biomass crops it may be anticipated that the long season, complex flux of rhizome depletion and filling, canopy closure and late season stem growth abortion and senescence makes simple associations difficult to uncover. This largely proved to be the case although some trends were identified as discussed below.

Across different years and explanatory variables the correlation between MaxGR and Duration was always highly or moderately significant and negative for Hy2. The trend was similar to *Mxg* when derived using factors Jd, GI and PTQ but became insignificant when derived from °Cd. In Hy2 the inverse correlation suggested that if the growth rate per unit temperature was higher the more Duration per unit temperature decreased, whereas in *Mxg* growth rate and Duration per unit temperature could both increase. When Duration was split in two further phases the period between StartLogG and DayMaxGR varied significantly across years in both genotypes parameterized with all four factors. However the period between DayMaxGR and EndLogG varied significantly except when parameterized by °Cd in *Mxg* (Figure 2). This may suggest that the factors that limit the extent of the logarithmic growth in rapidly growing plants of Hy2 do not similarly limit *Mxg*. We hypothesize that the consistency of the second period of Duration, only when calculated using accumulated temperature, indicated that in our experiment the logarithmic growth period of *Mxg* may not be limited by additional environmental factors but by developmental signals linked to temperature and that this illustrates one difference between the two high yielding *Miscanthus* genotypes. The factors limiting Hy2 appear more complex and occur earlier in the season whether measured as Jd or °Cd and probably represent the expected decline in canopy efficiency or nutrient and water mobilization within mature canopies. Drought studies of *Mxg* have often concluded it has an optimistic photophysiology in that it uses available water with little stomatal regulation (e.g. Clifton‐Brown & Lewandowski, 2000). In addition a study of three different *Miscanthus* types concluded that *Mxg* had exceptional radiation use efficiency (Davey et al., 2017) and our analysis is more in keeping with these experiments showing that *Mxg* is highly active in harvesting materials for growth and appears to be less limited than other similarly high yielding genotypes.

Genotypes with high MaxGR and shorter Duration may achieve the double benefit of high yield plus sufficient time for the crop to dry to low moisture content before harvest. In harvested biomass moisture content is an important compositional characteristic that affects safe storage and efficiency of processing (Clausen, 1994; Lewandowski et al., 2003). The moisture content of the crop was only correlated with Duration, the positive correlation indicated that longer Durations resulted in more moisture content of the harvested crop. This seems consistent with previous studies that have examined the effect of senescence on moisture content. For example in a diverse *Miscanthus* population delayed senescence or stay‐green was associated with higher moisture content (Robson et al., 2011). This need not necessarily be the case because longer duration could originate from earlier StartLogG; however, the longer duration genotypes Hy3 and Hy4 were also the latest to enter and leave the log growth phase therefore higher moisture content resulted from longer and/or later growth duration.

The use of growth curves to generate physiologically meaningful characteristics demonstrated the complex seasonal interactions between growth and biomass accumulation. Of particular note is a compensatory interaction between rate of growth and duration of growth that may limit overall yield. The analysis if applied to populations could identify genetic associations with maximum growth rates and the start and end of growth, identified here as important characteristics of yield. The growth curve characteristics could be used to predict how well adapted genotypes are to particular environments and to thereby parameterize a model to identify the optimum genotype for a particular location. The use of high yielding commercial genotypes showed that functional stem growth data are able to identify trends among superior germplasm. We identified differences in growth curve parameters associated with different meteorological factors that distinguished the commercial type, *Mxg*, from a high yielding competitor. Late season duration was limited more simply by temperature in *Mxg* which may explain in part the superior performance of the *Mxg* clone.
